# On the Vaccine Inoculation

**Published:** 1800-04

**Authors:** A. Huggan


### *On the Vaccine Inoculation; by Dr. Huggan.*

[ Continued from our laſt Number, pp. 241—245. ]

AS I am perfectly ſatisfied from the proof already before the public, of a perſon who has had the vaccine being thereby rendered unſuſceptible of variólous infection, I have not thought it neceſſary to inoculate any of my patients with the poiſon of the latter diſeaſe, having ſeen ſeveral of Mr. Stewart's, on whom the experiment was made, and with it, almoſt needleſs to add, the uſual effect.

Without preſuming to dictate, as I am not at all allowed to do ſo; from experience I venture to ſuggeſt, cold water, applied on lint, ſo as to keep the part conſtantly cool, as one of the likelieſt means of alleviating the pain from the inflammation tion of the arm, when that proves troublefome. This fymptom is undoubtedly, in a great meafure, occafioned by the punctures being made unneceffarily large, or when a thread is to be made ufe of in the inoculation; it was from this caufe chiefly, that one of my patients experienced fo much diftrefs from the inflammation; none of Mr. Stewart's had it in any greater degree than commonly follows the variolous incifion, nor any others of my own.

The method of performing this inoculation, from the difficulty that fome have experienced in being able to communicate the vaccine infection, has defervedly engaged the attention of medical men. Mr. Ring, with fome propriety, difapproves of Dr. Jenner's method, as tedious; his own, though certainly lefs exceptionable, is perhaps, itfelf, not the beft. (Medical and Phyfical Journal, Vol. II. p. 29.) As the method propofed by Mr. Ring, feems to lean to a theory of the manner in which the poifon produces the difeafe, viz. that by abforption, and which feems to be countenanced by the opinions of moft writers on the fubject, I fhall take the liberty of briefly ftating a few reafons, why it does not appear to be juft.

The power of the abforbents, in the healthy ftate of the body, and when their own action is unimpaired by general or local difeafe, is, as has been repeatedly proved, doubtlefs very great. Individually, however, their motion is apt to become irregular, or to be fufpended entirely, in confequence of external violence, or local difeafe. The action of thefe veffels, like that of the heart and blood veffels, being entirely independent of volition, is, like theirs alfo, liable to be affected by any fudden or violent agitation of the mind; thus, the faliva of a man in a fit of anger becomes vifcid, and his mouth dry; and alfo, when a perfon faints from fear, or any other depreffing paffion, we obferve feveral parts of the body to be covered with a cold clammy moifture, which is occafioned by the abforbents, (whofe motion is thus become retrograde) difgorging their contents, like the ftomach, whofe motion is often inverted alfo at the fame time. The affociated action of the parts, compofing the fanguiferous fyftem, being evidently greater than that of the lymphatics, yet from the irregular or fufpended action of a few of the fmaller blood veffels, there is no obvious change in the circulation; of courfe, the function of abforption will not be affected at all by the fimilar occurrences in a few of the abforbents.

When a vein, which in its ftructure, as well as in its functions, refembles an abforbent, is wounded, it either becomes paralyzed, and the blood, by its own gravity, efcapes from the wound, or its motion is inverted, and the blood flows backward; this latter circumftance fometimes proves fo troublefome in in great operations, that surgeons are obliged to secure the bleeding veins as well as the arteries.

The lachrymal ducts, when their extremities are inflamed, lose their power of motion, and the tears flow over the cheeks.

Now, in inoculation, the necessary puncture, or scratch, must be made with a light hand indeed, if one or more lymphatics, (provided any of them are in the way) are not wounded; hence, it may be presumed, that they will either be deprived of the power of motion, or that it will be inverted. If, however, any of the matter be left behind in inoculation, a circumstance not at all necessary, it will be washed out immediately by the blood, which, in but rare instances, does not issue from the puncture.

Independent, however, of all reasoning, this opinion seems to be fully refuted by the celebrated Mr. Hunter's decisive experiment, of cutting out the inoculated part of a patient's arm, after the specific inflammation had commenced, thereby preventing the constitutional disease.

As it is probable, therefore, that the power of action of such lymphatics as are wounded in inoculation, or become inflamed by "continuous sympathy," and whose canals seem thereby to be obliterated during the formation of the primary pustule, is suspended, if not entirely lost, the matter will not likely find an inlet into the body through this channel.

If, however, it could be proved to a certainty, that the vaccine, (as well as the variolous) virus, must necessarily be carried through the mass of circulating fluids,—why have recourse for that purpose to an operation, which, in its consequences, frequently becomes so distressing to the patient?

We have ascertained the power of the absorbents to be so great, as to take up not only such animal secretions as hog's lard, &c. but even grosser substances, as, opium, metallic calces, &c. and from whence we may fairly conclude, that they possess an equal power over matter of finer or more minute component parts, as the virus of either of these two diseases, and which may easily be applied in such forms, as to be absorbed with certainty, especially as it is admitted, that "a very small quantity of most morbid poisons," (as the variolous, vaccine, &c.) "however much diluted by the fluids of the human body, or by simple water, is as capable of exciting disease,—as matter containing the poison in its most concentrated state." (Medical and Physical Journal, Vol. I. p. 452.) But the fact is, that neither the variolous, nor vaccine diseases, can be excited in this way; nor is it easy to imagine, how the blood can become susceptible to the action of any poison, unless, indeed, we dream with Mr. Hunter about its possessing vitality, &c.

When

When mercury, fulphur, &c. are conveyed into the body by friction, we obferve, that they are thrown out again almoft immediately by the common excretories. What proof is there to the contrary, that any poifon, or other foreign matter, is lefs inoffenfive with refpect to the human fluids, or that it can be retained for a greater length of time?

The moft fatisfactory explanation of the manner in which the vaccine (as well as the variolous) virus, produces difeafe, is, that it acts primarily and folely upon the nervous fyftem, and in confequence of which only it is, that any change in the ftate of the blood takes place. The fpecific inflammation of the part to which the poifon is applied, conftitutes, we fuppofe, the firft or introductory link of that catenation of motions which it is the peculiar property of that poifon to excite. Hence, in inoculation, a flight puncture, made with the point of a lancet, fo as to wound a fentient extremity, is all that is neceffary, of which the moft unequivocal proof is, the flowing of the blood. This is the method which I have always followed, and have feldom failed in this way of communicating the infection. The infertion of a piece of thread, impregnated with matter, in the arm, is a very uncertain method of inoculating.

The ftate of the matter is another circumftance deferving attention; Dr. Pearfon directs it to "be taken from the arm of a patient, and immediately applied in its fluid ftate," and that we "muft not wait for fuppuration, and, indeed, if that comes on, the matter cannot be depended upon."——"The matter is in an efficacious ftate from the eighth to the eleventh day generally." * Though I have not always failed, when I have not had it in my power to avail myfelf of thefe hints, yet, from the little experience which I have had in inoculation with vaccine matter, I am fully convinced, that they cannot be too ftrictly complied with; as from inattention to the ftate of the matter, fome of my patients did not ficken before the eleventh or twelfth day, and on the arm of one of them, the fpecific inflammation did not commence before the ninth. That the matter may not lofe any of its active properties, it fhould be preferved in a vial quite dry, and clofely ftopped, or in hydrogen or nitrogen gas, as Dr. Pearfon recommends. The inoculation, though from the fimple manner in which it can be performed, appears fo trivial a circumftance, yet, it may occafionally be of the laft importance to the feelings and fafety of an individual, as well as the reputation of the furgeon, that

 it

---

* In a letter which I had the honour of receiving from Dr. Pearfon.

it be performed ſo as to inſure the infection taking place. A patient of mine was inoculated twice by means of a thread inſerted into each arm, without effect, and a third time by puncture; on the following day the ſmall-pox appeared.

D. C. ſeventeen months old, was inoculated by puncture, with vaccine matter taken from a puſtule in the ſtate of ſuppuration, and dried; on the ſixth day the firſt inflammation had not ſubſided, on the ſeventh it was leſs, and on the eighth had almoſt entirely diſappeared. He was again inoculated (25th Dec.) with recent matter, in its fluid ſtate; on Saturday the infection had evidently taken place: that evening the child, who had been ſeemingly unwell the day before, was taken very ill, and on Sunday the ſmall-pox appeared, of the confluent kind; on Monday, the puſtules in each arm, from the inoculation, coming forward; on the 31ſt day they contain matter; the child's body is covered with petechial ſpots; not a hope of his recovery is entertained. It will, perhaps, in general be found, that if the firſt inflammation continues beyond the fifth day, the infection will not take place. Now, from the event of this caſe, as the child is ſince dead, which might, perhaps, have been prevented, if the matter uſed in the firſt inoculation had been taken earlier from the patient, and in a more fluid ſtate, as the child was not unſuſceptible of the diſeaſe, the propriety of Dr. Pearſon's ſuggeſtions on this part of the ſubject will appear evident. I am,

GENTLEMEN,

Your moſt obedient ſervant,

A. HUGGAN.

